# Neuroprotective Effects of Cryptotanshinone in a Direct Reprogramming Model of Parkinson’s Disease

**DOI:** 10.3390/molecules25163602

**Published:** 2020-08-07

**Authors:** Joo-Eun Lee, Hyuna Sim, Hee Min Yoo, Minhyung Lee, Aruem Baek, Young-Joo Jeon, Kang-Sik Seo, Mi-Young Son, Joo Seog Yoon, Janghwan Kim

**Affiliations:** 1 Stem Cell Convergence Research Center, Korea Research Institute of Bioscience and Biotechnology (KRIBB), Daejeon 34141, Korea; jooeunlee@kribb.re.kr (J.-E.L.); hyunasim@kribb.re.kr (H.S.); minhyung@kribb.re.kr (M.L.); areumbaek@kribb.re.kr (A.B.); jeonyj@kribb.re.kr (Y.-J.J.); myson@kribb.re.kr (M.-Y.S.); 2 Department of Functional Genomics, KRIBB School of Bioscience, University of Science and Technology, Daejeon 34113, Korea; 3 Group for Biometrology, Korea Research Institute of Standards and Science (KRISS), Daejeon 34113, Korea; hmy@kriss.re.kr; 4 Huen Co., Ltd., Gwanggyo Business Center 5F (#508), 156, Gwanggyo-ro, Yeongtong-gu, Suwon 16506, Korea; ksseo@huenbio.com

**Keywords:** Parkinson’s disease, cryptotanshinone, disease modeling, mitochondrial dysfunction, antioxidant

## Abstract

Parkinson’s disease (PD) is a well-known age-related neurodegenerative disease. Considering the vital importance of disease modeling based on reprogramming technology, we adopted direct reprogramming to human-induced neuronal progenitor cells (hiNPCs) for in vitro assessment of potential therapeutics. In this study, we investigated the neuroprotective effects of cryptotanshinone (CTN), which has been reported to have antioxidant properties, through PD patient-derived hiNPCs (PD-iNPCs) model with induced oxidative stress and cell death by the proteasome inhibitor MG132. A cytotoxicity assay showed that CTN possesses anti-apoptotic properties in PD-hiNPCs. CTN treatment significantly reduced cellular apoptosis through mitochondrial restoration, such as the reduction in mitochondrial reactive oxygen species and increments of mitochondrial membrane potential. These effects of CTN are mediated via the nuclear factor erythroid 2-related factor 2 (NRF2) pathway in PD-hiNPCs. Consequently, CTN could be a potential antioxidant reagent for preventing disease-related pathological phenotypes of PD.

## 1. Introduction

Parkinson’s disease (PD) is a common progressive movement disorder that is represented by the loss of dopaminergic (DA) neurons in the substantia nigra (SN) and is characterized by tremors, bradykinesia, postural instability, and non-motor symptoms [1]. Multiple reports suggest that the cause of PD involves alterations in diverse cellular processes, such as mitochondrial functions, DA regulation, calcium homeostasis, proteostasis, and autophagy-lysosomal pathways [2,3]. Although the exact mechanisms of PD are still unclear, previous studies have shown that cellular protection through modulation of mitochondria would be effective in treating PD [4,5,6].

Mitochondrial dysfunction is linked to the pathogenesis of PD. The administration of 1-methyl-4-phenyl-1,2,3,6-tetrahydropyridine (MPTP) or rotenone, which are mitochondrial complex I inhibitors, replicates PD phenotypes, including subsequent respiratory inhibition, ATP depletion, oxidative stress, and neuro-inflammation in various models of PD [7,8,9]. In particular, clinical studies have suggested that mitochondrial activity is decreased in the SN of PD patients [10,11,12,13]. In addition, peroxisome proliferator-activated receptor γ coactivator-1alpha (*PGC1α*), an essential regulator of mitochondrial biogenesis, was downregulated in PD patients [14]. Based on these studies, it is becoming evident that mitochondrial dysfunctions are contributory elements in the pathogenesis of PD.

Genetic factors, as well as environmental factors, are considered to be involved in the mitochondrial-mediated pathogenesis of PD progression. Mutations in genes associated with inherited PD, such as *PARK2* (Parkin), *PINK1*, *PARK7* (*DJ-1*), alpha-synuclein (*SNCA*), *GBA1*, and leucine-rich repeat kinase 2 (*LRRK2*), have been observed to be related to mitochondrial homeostasis [3,7,10,11,15]. Among them, pathogenic mutations of *LRRK2* induce inherited PD, and neurodegenerative phenotypes of *LRRK2* mutants are correlated with an increase in oxidative stress [16,17]. The Gly2019Ser (G2019S) mutation of *LRRK2* is the most well-studied mutation in reprogramming-based disease models concerning mitochondrial functions. Moreover, it has been suggested that the *LRRK2* G2019S mutation reduces the antioxidant defense mechanisms of mitochondria through elevated levels of reactive oxygen species (ROS) [5]. With antioxidant reagents exhibiting protective effects on DA neurons in animal PD models, the inhibition of oxidative stress through the induction of mitochondrial biogenesis could potentially be a promising therapeutic mediation against PD [18,19].

Cryptotanshinone (CTN) is extracted from the dried roots (named Danshen) of *Salvia miltiorrhiza Bunge*, a member of the Lamiaceae family [20]. CTN is known to exhibit diverse biological effects, such as antioxidant activity, anti-cancer, and anti-inflammation effects [21,22,23,24]. CTN could potentially be utilized for therapeutic applications for neurodegenerative disorders due to its characteristics, such as its low molecular weight and its capacity to penetrate the blood–brain barrier (BBB) through lipophilicity. Recently, CTN was used to facilitate the significant recovery of rodent PD models from MPTP-induced oxidative stress injury [25]. Therefore, CTN treatment could potentially play an important role in alleviating neurodegenerative phenotypes in PD patients. However, rodent models may not sufficiently represent the correlation of aging process, genetic factors, and environmental insults in PD patients [26,27]. Thus, it is appropriate to incorporate human-based models that exhibit the complex pathological phenotypes of PD to evaluate the effectiveness of CTN for PD.

In our previous report, we developed human-induced neuronal progenitors (hiNPCs) from fibroblasts of PD patients harboring the *LRRK2* G2019S mutation and healthy control subjects [28]. Additionally, directly converted neural cells are regarded as valuable sources of disease modeling for late-onset neurodegenerative disorders [29,30]. Accordingly, we used PD patient-derived hiNPCs as an in vitro PD model to examine the effect of CTN in this study. We found that the cellular protective effect of CTN consequently mediated via the upregulation of nuclear factor erythroid 2-related factor 2 (*NRF2*)-mediated antioxidant molecules.

## 2. Results and Discussion

In this study, we used directly reprogrammed hiNPCs to examine the effects of CTN [20]. There are various types of cellular stressors available to establish the toxin-based protocols for modeling PD [16]. Among them, MG132, a cell-permeable proteasome inhibitor, has been used to induce ROS and mitochondrial dysfunction, ultimately promoting apoptotic cell death in in vitro and in vivo models of PD [31,32,33]. Predictably, hiNPCs from PD patients exhibited disease phenotypes following treatment with MG132. To investigate the protective role of CTN on hiNPCs from PD patients, the CTN treatment was conducted before the stress condition and the pathological phenotypes of the cells were analyzed. As a result, CTN exhibited neuroprotective effects on hiNPCs from PD patient in terms of mitochondria-mediated apoptotic cell death. These results are consistent with previous findings which reported that CTN prevented oxidative stress injury in MPTP-induced mouse PD models [25].

### 2.1. MG132-Induced In Vitro Model for PD Using hiNPCs from PD Patient

**Figure 1:**
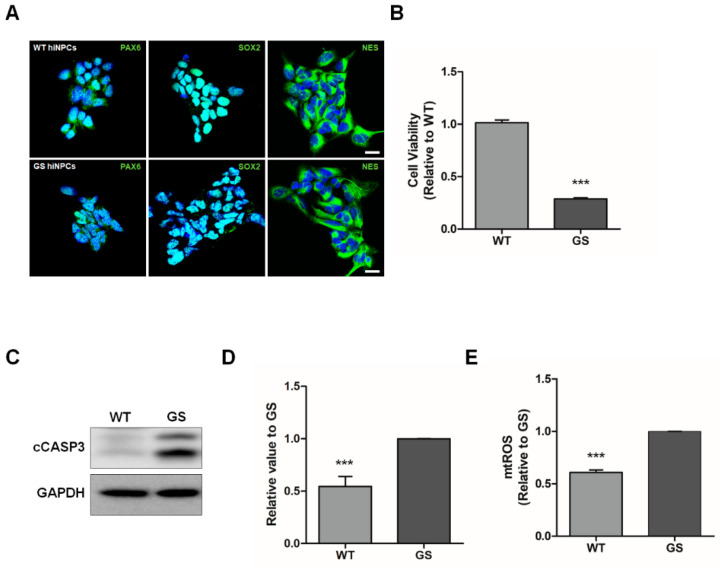
Parkinson’s disease (PD) modeling using patient-derived human-induced neuronal progenitor cells (hiNPCs). (**A**) Immunostaining of neural progenitor cell markers in hiNPCs (scale bars are 20 µm). (**B**) Cell viability assay with MG132 treatment. (**C**) Representative Western blot for cleaved caspase-3 (cCASP3) in hiNPCs treated with MG132. Glyceraldehyde 3-phosphate dehydrogenase (GAPDH) was used as an internal control. (**D**) Quantification of cCASP3 normalized over GAPDH by densitometry analysis. (**E**) Mitochondrial reactive oxygen species (mtROS) of MG132-treated hiNPCs stained with MitoSOX. WT, healthy control-derived hiNPCs; GS, familial LRRK2 G2019S PD patient-derived hiNPCs. Data are mean ± standard error of means (SEM) of at least three independent experiments. *p*-values were analyzed using the unpaired two-tailed Student’s *t*-test (*** *p* < 0.001).

The hiNPCs from fibroblasts of PD patients and healthy control subjects were generated as described in our previous report [28]. MG132, a cell-permeable proteasome inhibitor, was used to mimic the pathological phenotypes of PD [16,31,32]. To determine the protective effect of CTN in an in vitro model for PD, we used hiNPCs from fibroblasts of familial PD patients harboring the *LRRK2* G2019S mutation (GS) and of healthy controls (WT). The hiNPCs exhibited neural progenitor cell markers such as paired box 6 (PAX6), SRY-Box transcription factor 2 (SOX2), and nestin (NES) (Figure 1A). The pathological phenotypes of PD were induced through MG132 treatment (10 μM) for 24 h. It has long been known that pathogenic *LRRK2* mutations are related to mitochondrial dysfunctions and increased stress susceptibility to oxidative stress [3]. Accordingly, we initially performed a cell viability assay (Figure 1B) and Western blotting (Figure 1C,D) to evaluate cellular apoptosis. As a result, GS hiNPCs exhibited significantly reduced cell viability and increased expression of cleaved caspase-3 (cCASP3) compared to WT hiNPCs. Furthermore, we observed that GS hiNPCs exhibited mitochondrial dysfunctions after treatment with MG132, such as increased mitochondrial ROS compared to WT hiNPCs (Figure 1E). These results suggest that directly reprogrammed hiNPCs from PD patients could be an appropriate in vitro model for PD through MG132 treatment.

### 2.2. Cytoprotective Effects of CTN on the In Vitro PD Model

**Figure 2:**
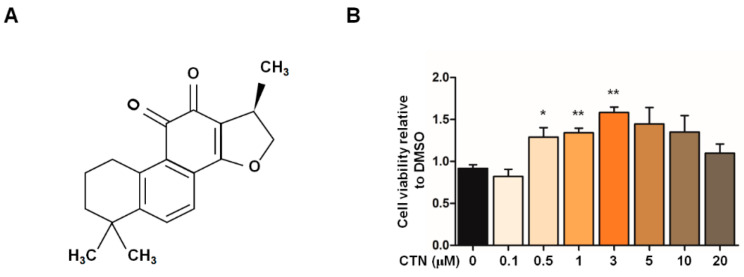
Cytoprotective effects of cryptotanshinone (CTN) on MG132-induced cellular stress in GS-hiNPCs. (**A**) Chemical structure of CTN from the root of *Salvia miltiorrhiza Bunge* (Danshen). (**B**) Cytoprotective effects of CTN on GS hiNPCs with the MG132-induced stress condition. Data are mean ± standard error of means (SEM) of at least three independent experiments. *p*-values were analyzed using the unpaired two-tailed Student’s *t*-test (* *p* < 0.05, ** *p* < 0.005).

To confirm whether CTN (Figure 2A) exhibits neuroprotective effects in our PD model, we treated GS hiNPCs with CTN for 24 h in a dose-dependent manner, prior to the stress condition with MG132 (Figure 2B). CTN (0.5–3 μM) showed significant cytoprotective effects on GS hiNPCs.

### 2.3. Cell Death Attenuation by CTN

**Figure 3:**
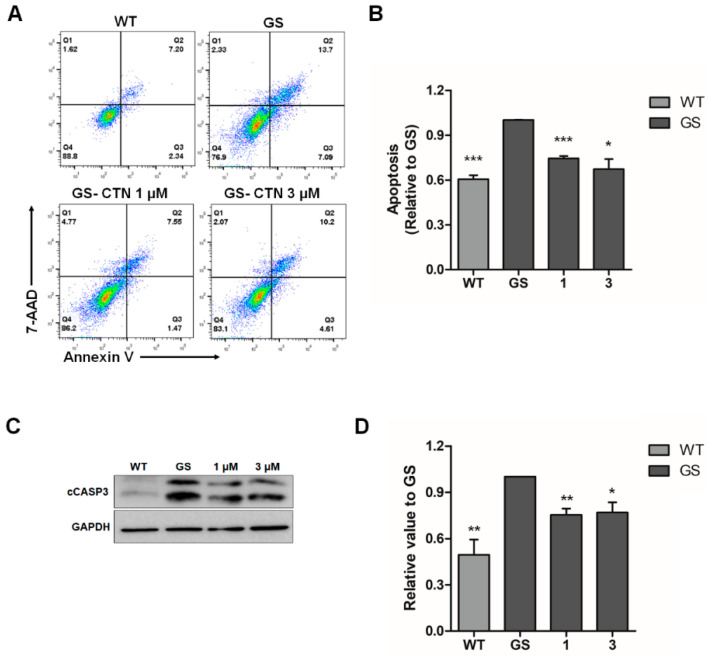
Effects of CTN on MG132-induced apoptosis in GS hiNPCs. (**A**) MG132-induced apoptotic cell death and recovery by CTN treatment were evaluated through Annexin V/7-AAD staining. (**B**) Quantification of apoptotic cells by flow cytometry. (**C**) Cell lysates from CTN-treated hiNPCs immunoblotted with the apoptosis marker cCASP3. GAPDH was used as an internal control. (**D**) Quantification of Western blots for cCASP3 normalized over GAPDH. Data are mean ± standard error of means (SEM) of at least three independent experiments. *p*-values were analyzed using the unpaired two-tailed Student’s *t*-test (* *p* < 0.05, ** *p* < 0.005, *** *p* < 0.001).

To further evaluate the effectiveness of CTN on our in vitro PD model, cellular stress was induced by MG132 to mimic PD pathologies in GS hiNPCs and WT hiNPCs, following treatment with dimethyl sulfoxide (DMSO) or CTN (1, 3 μM) for 24 h. The cells were stained with Annexin V and 7-AAD dyes, which are used to quantify apoptotic cell death (Figure 3A,B). GS hiNPCs showed significantly increased apoptotic cell death compared to WT hiNPCs in the MG132-induced stress condition. In addition, CTN-treated GS hiNPCs exhibited significantly attenuated early and late apoptosis within 24 h of treatment. The main markers of apoptosis in hiNPCs in response to CTN (1, 3 μM) treatment were also analyzed through Western blotting (Figure 3C,D). We found that treatment with CTN significantly downregulated the expression of the apoptotic protein cCASP3 in GS hiNPCs. Therefore, we confirmed that MG132-induced apoptotic cell death in GS hiNPCs was reduced by CTN.

### 2.4. Recovery of Mitochondrial Functions by CTN

**Figure 4:**
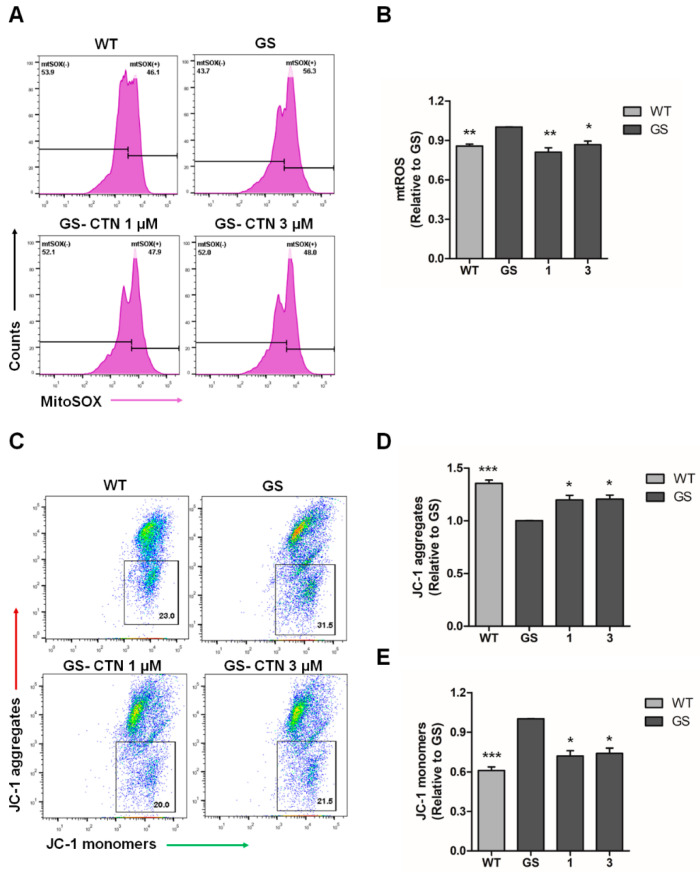
Evaluation of the regulation of mitochondrial functions by CTN treatment under MG132-induced oxidative stress. (**A**) MG132-induced increases in mitochondrial reactive oxygen species (ROS) levels and restoration by CTN treatment as analyzed by MitoSOX-based flow cytometry. (**B**) Quantification of mtROS levels. (**C**) Evaluation of mitochondrial membrane potential (MMP) through JC-1 aggregates and JC-1 monomer fluorescence intensity through flow cytometry. (**D**) Quantification of JC-1 aggregates population. (**E**) Evaluation of JC-1 monomers. Data are mean ± standard error of means (SEM) of at least three independent experiments. *p*-values were analyzed using the unpaired two-tailed Student’s *t*-test (* *p* < 0.05, ** *p* < 0.005, *** *p* < 0.001).

Treatment with proteasome inhibitors leads to mitochondria-mediated cascades including mitochondrial depolarization, formation of mitochondrial ROS, and activation of caspase-3 [34,35,36]. Furthermore, ROS production can be initiated by mitochondrial dysfunction and can subsequently induce cell death in PD patients [37,38]. The *LRRK2* G2019S mutation can particularly cause defects in mitochondrial functions and increase the generation of ROS [39,40]. Moreover, PD patient-derived cells harboring the *LRRK2* G2019S mutation exhibited decreased mitochondrial membrane potential [33]. Thus, we investigated whether CTN regulates mitochondrial alterations in our in vitro PD model (Figure 4). We found that the total and mitochondrial ROS levels of GS hiNPCs were increased compared to WT hiNPCs, which were restored through CTN treatment (1, 3 μM) for 24 h (Figure S1, Figure 4A,B). Next, the effectiveness of CTN on mitochondrial membrane potential (MMP) was investigated by probing JC-1 fluorescence intensity [41]. The MMP depolarization properties and early apoptotic populations of GS hiNPCs and WT hiNPCs were detected through JC-1 aggregates (Figure 4C,D) and JC-1 monomer intensity (Figure 4C,E), respectively. The results show that GS hiNPCs treated with CTN (1, 3 μM) for 24 h showed significant MMP recovery and fewer early apoptotic cells. These results suggest that the CTN potentially protects cells through improvement of mitochondrial functions in our hiNPC model for PD.

### 2.5. Induction of NRF2-Mediated Oxidative Stress Response by CTN

**Figure 5:**
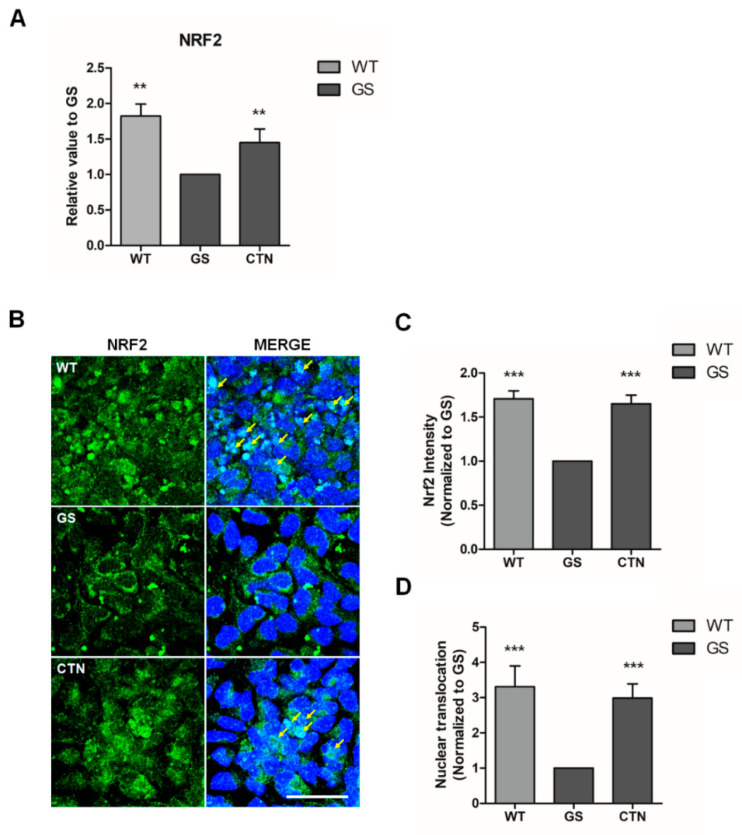
Expression and sub-cellular localization of nuclear factor erythroid 2-related factor 2 (*NRF2*) in an in vitro PD model. (**A**) mRNA expression of *NRF2*—the master regulator of antioxidant responses. (**B**) Immunofluorescence analysis of NRF2 labeled by Fluorescein (FITC, green). Cell nuclei were stained with Hoechst33342 (blue). Scale bar is 20 µm. (**C**) Intensities of NRF2 expression. (**D**) Quantification of NRF2 nuclear translocation. Data are mean ± standard error of means (SEM) of at least three independent experiments. *p*-values were analyzed using the unpaired two-tailed Student’s *t*-test (** *p* < 0.005, *** *p* < 0.001).

The results above indicate that CTN restores mitochondrial ROS and secures cells from apoptosis. Following this, we further investigated molecules involved in this protective process. We found that the mRNA expression of *NRF2*, which is known to induce the expression of antioxidants as well as cellular protective molecules [42,43,44], was significantly downregulated in GS hiNPCs compared to WT hiNPCs (Figure 5A). These results are consistent with the previous reports in various PD models [45,46,47]. After treatment with CTN (3 μM) for 24 h, the mRNA expression levels of *NRF2* were upregulated significantly in GS hiNPCs. We further analyzed the distribution of NRF2 through immunofluorescence (Figure 5B–D). The fluorescence intensity of *NRF2* had decreased in GS hiNPCs (Figure 5B,C) and the NRF2 expression was mainly allocated to the cytoplasm (Figure 5B,D). After treatment with CTN (3 μM), the NRF2 expression levels recovered in GS hiNPCs and the sub-cellular distribution of NRF2 were reversed from the cytoplasm to the nucleus (Figure 5B–D). These results suggest that CTN could be a potent neuroprotective agent that regulates the *NRF2* pathway for the treatment of PD patients.

### 2.6. Expression of Antioxidative and Mitochondrial Biogenesis Molecules by CTN

**Figure 6:**
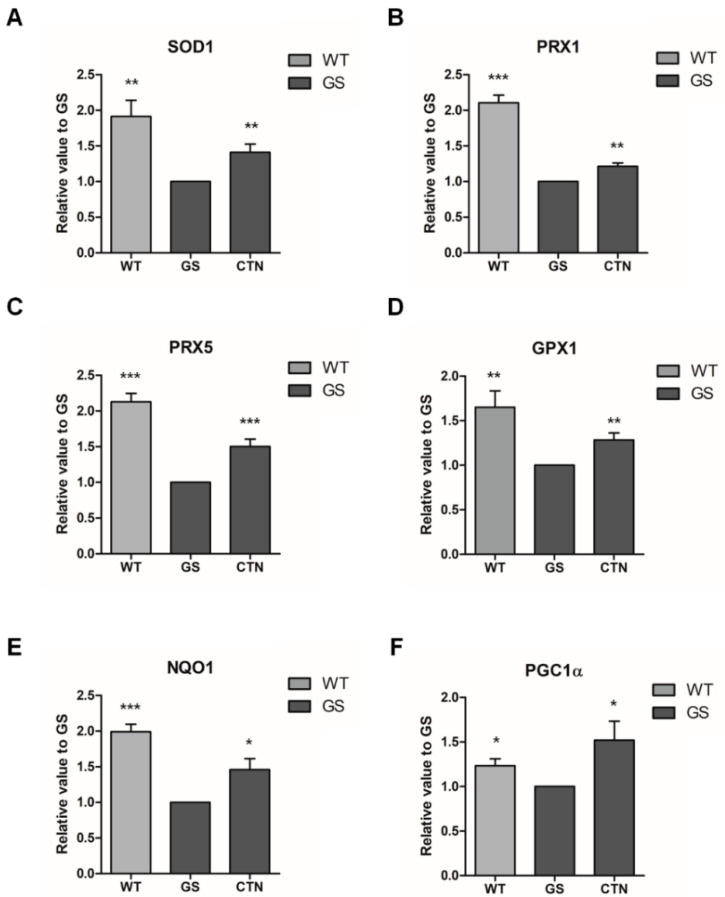
mRNA expression of *NRF2*-mediated cytoprotective molecules. Antioxidant molecules, such as (**A**) superoxide dismutase 1 (*SOD1*), (**B**) peroxiredoxin 1 (*PRX1*), (**C**) peroxiredoxin 5 (*PRX5*), (**D**) glutathione peroxidase 1 (*GPX1*), and (**E**) the phase II antioxidant enzyme NAD(P)H: quinone oxidoreductase 1 (*NQO1*) were analyzed in WT hiNPCs, GS hiNPCs, and GS hiNPCs treated with CTN (3 μM) for 24 h followed by MG132 treatment (10 μM, 24 h). In addition, the mitochondrial biogenesis marker (**F**) peroxisome proliferator-activated receptor γ coactivator-1alpha (*PGC1α*) was analyzed. Data are mean ± standard error of means (SEM) of at least three independent experiments. *p*-values were analyzed using the unpaired two-tailed Student’s *t*-test (* *p* < 0.05, ** *p* < 0.005, *** *p* < 0.001).

To further investigate the antioxidative effects of CTN, mRNA expression levels of *NRF2*-mediated cytoprotective genes, such as superoxide dismutase 1 (*SOD1*), peroxiredoxin 1 (*PRX1*), peroxiredoxin 5 (*PRX5*), glutathione peroxidase 1 (*GPX1*), and NAD(P)H: quinone oxidoreductase 1 (*NQO1*) [44,48,49,50] were analyzed in GS hiNPCs through real-time PCR (Figure 6A–E). The results demonstrate that there were significant increases in the expression levels of all of the aforementioned genes following CTN treatment in GS hiNPCs. We also evaluated the mRNA expression levels of peroxisome proliferator-activated receptor γ coactivator-1alpha (*PGC1α*) [51], which is known as a marker of mitochondrial biogenesis (Figure 6F). Recent studies have shown that PGC1α functions are impaired in PD patients and the activation of PGC1α can protect cells from the loss of mitochondria [52,53,54,55]. In accordance with these reports, the mRNA expression levels of *PGC1α* in GS hiNPCs were found to be lower than in WT hiNPCs, and the expression levels recovered following CTN treatment (3 μM) for 24 h. Together, our results confirm that CTN modulates *NRF2*-mediated transcriptional activation of antioxidant signaling pathways in GS hiNPCs.

The establishment of improved models for PD is central to the investigation of disease-related characteristics. As primary cells affected by PD are difficult to obtain, alternative cell models such as PC12, MN9D, and SH-SY5Y have been used as in vitro models to study PD [56,57]. However, cell lines from different species or tumor-derived neuroblastoma have certain limitations in representing the pathophysiology of PD patients. More recently, human-induced pluripotent stem cell (iPSC) derived neural cells from PD patients are used to understand underlying mechanisms and develop therapeutic drugs [58]. Patient-specific iPSCs have remarkable potential to unveil novel insights in terms of the disease pathogenesis, although they are known to remain rejuvenated and lose aging signatures [59]. Meanwhile, directly reprogrammed neural cells from patient somatic cells are regarded as attractive models for PD as they retain metabolic, mitochondrial, epigenetic, and aging signatures [60,61,62]. Accordingly, we used PD patient-derived hiNPCs as an in vitro model to evaluate the effectiveness of CTN. Consistent with a previous report using an MPTP-induced in vivo PD model, CTN treatment restored pathological phenotypes in directly reprogrammed hiNPCs from PD patients. These results suggest that our in vitro PD model could be a valuable resource that allows researchers to mimic the characteristics of PD, and an appropriate tool that can be used to discover potential candidates for drug development.

## 3. Materials and Methods

### 3.1. Cell Culture

Fibroblasts of a PD patient harboring the *LRRK2* G2019S mutation (ND38262) and healthy control subjects (AG02261, GM01680) were purchased from the Coriell Institute (Camden, NJ, USA) and were reprogrammed to human-induced neural progenitor cells (hiNPCs) as described in our previous report [28]. The reprogrammed hiNPCs were cultured as follows: healthy control-derived (WT) hiNPCs and Parkinson’s disease patient-derived (GS) hiNPCs were plated on Geltrex^TM^ LDEV-Free Reduced Growth Factor Basement Membrane Matrix-coated plates with an hiNPC medium ((50% Advanced DMEM/F12 (Thermo Fisher Scientific, Waltham, MA, USA), 50% Neurobasal medium (Thermo Fisher Scientific, Waltham, MA, USA), 0.05% AlbuMAX^TM^ (Thermo Fisher Scientific, Waltham, MA, USA), N-2 Supplement (100X, Thermo Fisher Scientific, Waltham, MA, USA), B-27 Supplement minus vitamin A (50X, Thermo Fisher Scientific, Waltham, MA, USA), 2 mM Glutamax^TM^ (Thermo Fisher Scientific, Waltham, MA, USA), 0.11 mM 2-mercaptoethanol (Thermo Fisher Scientific, Waltham, MA, USA), 3 μM CHIR 99021 (Tocris Bioscience, Bristol, UK), 0.5 μM A 83-01 (Tocris Bioscience, Bristol, UK), and 10 ng/ml human LIF (PeproTech, Inc., Rocky Hill, NJ, USA)). The medium was replaced every second day. WT hiNPCs and GS hiNPCs were dissociated with Accutase cell detachment solution (Millipore Sigma, Burlington, MA, USA) every five to seven days. Our samples were determined to be exempt from Institutional Review Board (IRB) review.

### 3.2. Cell Viability Assay

Thirty thousand hiNPCs/well were seeded on a Geltrex^TM^ LDEV-Free Reduced Growth Factor Basement Membrane Matrix-coated 96-well TC-treated microplate. The ROCK Inhibitor Y-27632 (Tocris Bioscience, Bristol, UK) was added to the hiNPC medium with a concentration of 10 μM for 24 h. Various concentrations of cryptotanshinone (CTN, Sigma-Aldrich, St. Louis, MO, USA) or equal volumes of DMSO were added to the hiNPC medium for 24 h, followed by 10 μM MG132 (Sigma-Aldrich, St. Louis, MO, USA). Cell viability was measured using the EZ-Cytox enhanced cell viability assay kit (DoGenBio, Seoul, Korea) according to the manufacturer’s instructions. After 2 h, we measured absorbance using a Spectramax microplate reader (Molecular Devices, San Jose, CA, USA).

### 3.3. Flow Cytometry Analysis

Thirty thousand hiNPCs/well were seeded on a Geltrex^TM^ LDEV-Free Reduced Growth Factor Basement Membrane Matrix-coated 12-well TC-treated plate. The ROCK Inhibitor Y-27632 was added to the hiNPC medium with a concentration of 10 μM for 24 h. CTN (1, 3 μM) or equal volumes of DMSO were added to the hiNPC medium for 24 h, followed by 10 μM MG-132. For analysis, the hiNPCs were dissociated into single cells through incubation with Accutase cell detachment solution for 4 min. For the apoptosis assays, the cells were stained with Fluorescein (FITC) Annexin V and 7-amino-actinomycin D (7-AAD, BD Pharmingen^TM^, San Jose, CA, USA). For the detection of mitochondrial reactive oxygen species, the cells were treated with MitoSOX™ Red mitochondrial superoxide indicator (Thermo Fisher Scientific, Waltham, MA, USA), and JC-1 Dye (mitochondrial membrane potential probe) (Thermo Fisher Scientific, Waltham, MA, USA) was used to measure mitochondrial membrane potential. The cells were treated with the indicators for 15 min at 37 °C. Stained hiNPCs were detected using a BD FACSVerse^TM^ Flow Cytometer (BD Biosciences, San Jose, CA, USA) and the data were analyzed using the FlowJo software (version 10).

### 3.4. Western Blot Analysis

The cells were washed with Dulbecco’s phosphate-buffered saline (DPBS, Welgene, Kyungsan, Korea) and treated with a protein lysis buffer ((1% Triton X-100 (*v*/*v*), 1 mM phenylmethanesulfonyl fluoride (PMSF, Thermo Fisher Scientific, Waltham, MA, USA), 5 mM ethylenediaminetetraacetic acid (EDTA, Thermo Fisher Scientific, Waltham, MA, USA), and Xpert protease inhibitor cocktail solution (100X, GenDEPOT, Katy, TX, USA)). Total protein concentration was determined using protein assay dye reagent concentrate (Bio-Rad Laboratories, Inc., Hercules, CA, USA). An equal amount of protein was boiled at 95 °C for 5 min and loaded for SDS-PAGE. The proteins were transferred onto a Polyvinylidene fluoride (PVDF) membrane (Bio-Rad Laboratories, Inc., Hercules, CA, USA), which was pre-wetted in methanol for 3 min, using Wet/Tank Blotting Systems (Bio-Rad Laboratories, Inc., Hercules, CA, USA). The membrane was blocked with 5% skim milk (Difco^TM^ Skim Milk, BD Biosciences, San Jose, CA, USA) at room temperature for 30 min. We then incubated the membrane with primary antibodies (Table S2) overnight at 4 °C, followed by incubation with horseradish peroxidase (HRP)-conjugated secondary antibodies (Cell Signaling Technology, CST, Danvers, MA, USA). We used the ECL^TM^ Select Western Blotting Detection Reagent (GE Healthcare, Chicago, IL, USA) for the detection of HRP signals. Protein bands were acquired using an LAS-3000 imaging system (Fujifilm, Minato, Tokyo, Japan) and were quantified using the NIH Image J software.

### 3.5. Real-Time PCR for mRNA Quantification

Total RNA was extracted using the TRIzol^TM^ reagent (Thermo Fisher Scientific, Waltham, MA, USA) and was reverse-transcribed using an iScript cDNA synthesis kit (Bio-Rad Laboratories, Inc., Hercules, CA, USA) according to the manufacturer’s instructions. Quantitative real-time PCR (qRT-PCR) was performed using a 7500 Fast Real-Time PCR system (Applied Biosystems, Foster City, CA, USA). Glyceraldehyde 3-phosphate dehydrogenase (GAPDH) was used as an internal control gene. The primer sequences used in this study are listed in Table S1.

### 3.6. Immunofluorescence

The hiNPC culture medium was removed, and the cells were fixed in 4% paraformaldehyde for 10 min at room temperature (RT) then washed three times with Tris-Buffered Saline (TBS). The cells were permeabilized with 0.01% Triton X100 (sigma) for 10 min and blocked in 5% Bovine Serum Albumin (BSA) for 30 min at RT. This was followed by incubation overnight with the primary antibodies (Table S2) at 4 °C. The cells were then washed three times with Tris-Buffered Saline, 0.05% Tween_®_ 20 Detergent (TBST) and incubated with the secondary antibodies for 1 h at RT. The cells were again washed three times with TBST and stained with Hoechst for 10 min at RT. Images were acquired using a confocal microscope (ZEISS LSM 820, Zeiss, Germany).

### 3.7. Statistical Analysis

Statistical analysis was conducted using GraphPad Prism 5 (GraphPad Software, Inc., San Diego, CA, USA), and the values were set as mean ± standard error of means (S.E.M). The results were presented using the unpaired, two-tailed Student’s *t*-test. *p*-values < 0.05 were considered as statistically significant. All experiments were performed with three to five technical replicates.

## 4. Conclusions

In this study, we investigated the protective effects of CTN on directly reprogrammed PD patient-derived neural cells. CTN treatment restored mitochondrial dysfunctions as well as oxidative stress-induced apoptosis in GS hiNPCs. Furthermore, CTN treatment significantly upregulated the expression of *NRF2* and promoted the translocation of *NRF2* into the nucleus. Subsequently, *NRF2*-mediated transcriptional activation of cytoprotective enzymes, such as *SOD1*, *PRX1*, *PRX5*, *GPX1*, *NQO1*, and *PGC1α*, were stimulated by the CTN treatment. The enhancement of these antioxidant molecules may lead to the attenuation of pathological phenotypes in GS hiNPCs—an in vitro PD model. In conclusion, CTN could be developed into a potential therapeutic agent for preventing PD.

## Supplementary Materials

The following are available online, Table S1: List of primer sequences used in this study. Table S2: List of antibodies. Figure S1. Evaluation of total ROS following CTN treatment under MG132-induced oxidative stress. (A) MG132-induced oxidative stress increases total ROS levels and restoration following CTN treatment as analyzed by DCF2DA-based flow cytometry. (B) Quantification of total ROS.
