# Supplementary material for: Neuroprotective Effects of Cryptotanshinone in a Direct Reprogramming Model of Parkinson’s Disease
Source: Molecules. 2020 Aug 7;25(16):3602. doi: 10.3390/molecules25163602 (PMC7463464; doi:10.3390/molecules25163602)
Supplement: Supplementary file 1 [file molecules-25-03602-s001.pdf]

Supplementary Material

Table S1. List of primer sequences used in this study.

Target	F/R	Sequences
NRF2	F	TCC AGT CAG AAA CCA GTG GAT
	R	GAA TGT CTG CGC CAA AAG CTG
SOD1	F	GAA GGT GTG GGG AAG CAT TA
	R	ACA TTG CCC AAG TCT CCA AC
PRX1	F	AAG AAA CTC AAC TGC CAA GTG
	R	CAG CCT TTA AGA CCC CAT AAT
PRX5	F	CCA ATC AAG ACA CAC CTG CC
	R	TCT TGA GAC GTC GAT TCC CA
GPX1	F	CCA AGC TCA TCA CCT GGT CT
	R	TCG ATG TCA ATG GTC TGG AA
NQO1	F	GGT GGA GTC GGA CCT CTA TG
	R	ATA TCA CAA GGT CTG CGG CT
PGC1α	F	GTC ACC ACC CAA ATC CTT AT
	R	ATC TAC TGC CTG GAG ACC TT

Table S2. List of antibodies used in this study.

Target	Vender	Catalog number	Dilution
PAX6	Biolegend	901301	1:500
SOX2	Millipore	MAB4343	1:200
NESTIN	Biolegend	839801	1:200
NRF2	Santa Cruz Biotechnology, Inc.	sc-365949	1:100
Cleaved CASP3 (Asp175)	Cell signaling technology	9661	1:1000
GAPDH	Santa Cruz Biotechnology, Inc.	sc-47724	1:5000

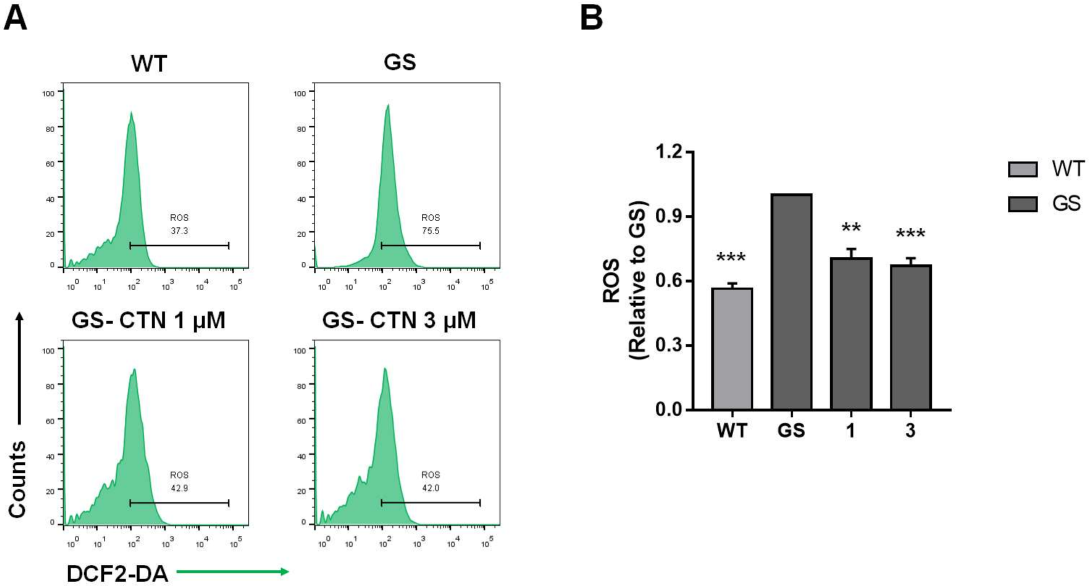

Figure S1. Evaluation of total ROS following CTN treatment under MG132-induced oxidative stress. (A) MG132-induced oxidative stress increases total ROS levels and restoration following CTN treatment as analyzed by DCF2DA-based flow cytometry. (B) Quantification of total ROS.
